# Evaluating TnP as a Potential Therapeutic Agent for Retinopathy in Zebrafish Models

**DOI:** 10.3390/ph18060840

**Published:** 2025-06-04

**Authors:** João Gabriel Santos Rosa, Jefferson Thiago Gonçalves Bernardo, Yolanda Álvarez, Breandán Kennedy, Carla Lima, Monica Lopes-Ferreira

**Affiliations:** 1 Immunoregulation Unit of the Laboratory of Applied Toxinology (CeTICs/FAPESP), Butantan Institute, São Paulo 05503-900, Brazil; joao.rosa.esib@esib.butantan.gov.br (J.G.S.R.); jefferson.bernardo@fundacaobutantan.org.br (J.T.G.B.); carla.lima@butantan.gov.br (C.L.); 2 UCD Conway Institute of Biomolecular and Biomedical Research and UCD School of Biomolecular and Biomedical Science, University College Dublin, D04 V1W8 Dublin, Ireland; yolanda.alvarez@ucd.ie (Y.Á.); brendan.kennedy@ucd.ie (B.K.)

**Keywords:** TnP, peptide, drug discovery, retinal damage, immune modulation, visual behavior, neuroprotection, diabetic retinopathy

## Abstract

**Background**: The retina plays a vital role in vision, and its impairment can cause significant visual deficits. Current retinal disease treatments range from conventional anti-inflammatory drugs to advanced anti-VEGF therapies and monoclonal antibodies. TnP, a novel synthetic peptide in preclinical development, has demonstrated therapeutic potential in chronic inflammatory conditions such as multiple sclerosis and asthma due to its immunomodulatory properties. Using zebrafish—which share significant genetic homology with humans—we investigated TnP’s effects on retinopathy models mimicking diabetic retinopathy (DR) through either cobalt chloride (CoCl_2_)-induced hypoxia or light-induced retinal damage (LIRD). **Methods**: We employed two retinal injury models (CoCl_2_-induced hypoxia and LIRD) and subjected them to TnP treatment, assessing the outcomes through visual–motor response testing and histological examination. **Results**: CoCl_2_ exposure impaired swimming activity, while light damage reduced the movement distance. Both models induced distinct retinal morphological changes. Although TnP failed to reverse most injury effects, it specifically restored the inner plexiform layer (IPL)’s thickness. **Conclusions**: Our findings suggest that TnP may enhance neuronal plasticity by promoting cell proliferation and synaptic connectivity. While showing promise as a therapeutic candidate for retinal and neurodegenerative disorders, TnP might achieve optimal efficacy when combined with complementary treatments.

## 1. Introduction

The retina plays a crucial role in sensory perception, and damage to its structure can lead to visual impairments, significantly affecting an individual’s quality of life. Conditions such as glaucoma, age-related macular degeneration (AMD), and diabetic retinopathy (DR) are major causes of vision loss and blindness, necessitating effective therapeutic interventions.

According to the World Health Organization (2019), 146 million people are affected by DR, with projections estimating that 95.4 million will suffer from glaucoma and 243.4 million from AMD by 2030. Globally, the costs associated with prevention and treatment are expected to exceed USD 16.8 billion.

Diabetic retinopathy, one of the most common complications of diabetes, leads to vision impairment and blindness due to retinal vascular damage [1,2]. Additionally, impaired photoreceptor function is associated with all stages of diabetic retinopathy [3].

Retinal degeneration often involves significant inflammatory components, and the inflammatory response in many retinal diseases is excessive, playing a key role in disease progression. Current treatments include anti-inflammatory drugs [4], but these can cause adverse effects such as osteoporosis, gastrointestinal disorders, and cardiovascular complications [5,6].

New therapeutic approaches, such as anti-VEGF medications, monoclonal antibodies targeting VEGF isoforms and Angiopoietin-3, and gene therapy tailored to specific diseases (reviewed in [7]), have emerged. However, these treatments remain largely inaccessible due to their high costs and technical delivery requirements, highlighting the need for more affordable and effective therapies for chronic inflammatory retinal diseases.

The TnP peptide, a synthetic compound consisting of 13 L-amino acids, has been patented in several countries, including Brazil. As a preclinical drug candidate, TnP has shown promise in treating chronic inflammatory diseases such as multiple sclerosis and asthma. Its anti-inflammatory effects have been demonstrated in murine models of experimental autoimmune encephalomyelitis (EAE) [8,9] and OVA-induced acute asthma [10].

TnP’s potential in controlling chronic inflammation stems from its ability to modulate immune cell activity, both systemically and locally, in tissues such as the central nervous system (CNS) and lungs. This suggests that it may also be effective in retinopathies, which involve chronic inflammation. Growing evidence links inflammation to diabetes-related retinal damage, associating inflammatory responses with pathological angiogenesis and neuro-glial degeneration [11,12].

Despite its therapeutic potential, the precise mechanism of TnP’s action remains unclear. Cellular receptors are likely key mediators of its effects, as peptide–protein interactions play a fundamental role in cellular functions. Bioinformatics analyses by Lima, Eto, and Lopes-Ferreira (2022) [13] suggest that TnP may interact with RGD-dependent integrins, potentially regulating leukocyte migration.

The retina and its neurovascular unit are highly conserved between humans and zebrafish, with both species sharing similar vascular development pathways and gene expression patterns [3,14,15]. Zebrafish offer several advantages as a research model, including 70% genetic similarity to humans (rising to 80% for disease-related genes) [3,15], optical transparency during embryonic stages, and cost-effective maintenance.

In zebrafish, retinal neurogenesis begins with ganglion cell differentiation at around 28 h post-fertilization (hpf), followed by amacrine, horizontal, and Müller glial cell development. By 48 hpf, retinal lamination is mostly complete, with opsins, rod cells, and cone photoreceptors becoming detectable. Functional synaptic transmission between photoreceptors and second-order neurons is established by 120 hpf [16].

The zebrafish retina has a well-organized laminar structure, consisting of three nuclear layers: the outer nuclear layer (ONL, containing photoreceptor cell bodies), the inner nuclear layer (INL, housing amacrine, bipolar, horizontal, and Müller glial cells), and the ganglion cell layer (GCL, containing ganglion cell bodies). These layers are separated by the outer and inner plexiform layers (OPL and IPL), where synaptic connections occur (reviewed in [17]).

Previous studies on TnP in zebrafish have explored its therapeutic index, safety, toxicology, tissue distribution (including CNS penetration) [18], and potential mechanisms of action [19]. Building on this foundation, our study aimed to evaluate TnP’s therapeutic potential in retinopathy using zebrafish models of retinal degeneration induced by hypoxia or light damage.

## 2. Results

### 2.1. Recapitulation of Retinopathy in Zebrafish Larvae

**Figure 1:**
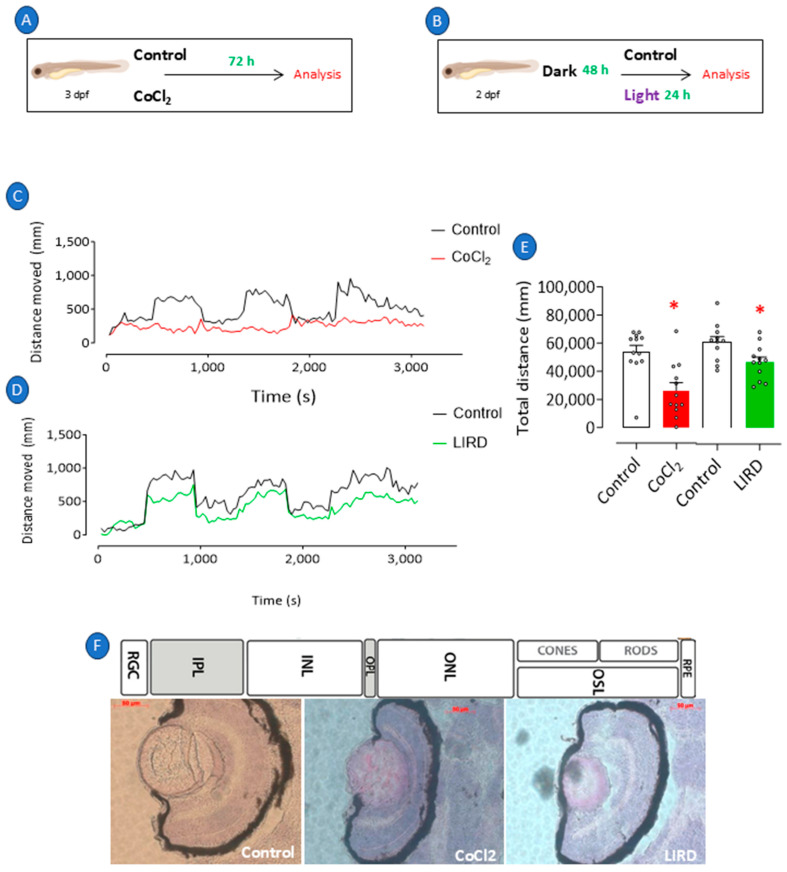
Retinopathy in zebrafish larvae. Retinopathy was induced in 3 dpf larvae through exposure to E2 0.5x medium at 28 °C with 0.5 mM cobalt chloride (CoCl_2_) for 72 h (**A**). Independent group of 3 dpf larvae was exposed to intense light at 8000 lux for 24 h (LIRD) (**B**). Larvae maintained at 28 °C in E2 0.5x medium only and under natural light were considered negative controls. Immediately after stimulation, all groups of larvae were evaluated for their visual–motor response (**C**,**D**) and distance traveled (**E**) and were subsequently killed and processed for histological analysis and H&E staining (**F**). * *p* < 0.05 compared to control group. Bars represent mean ± SEM. Dots represent individual data points. RGL: retinal ganglion layer; IPL: inner plexiform layer; INL: inner nuclear layer; OPL: outer plexiform layer; ONL: outer plexiform layer; OSL: outer segment layer; RPE: retinal pigment epithelium.

**Figure 2:**
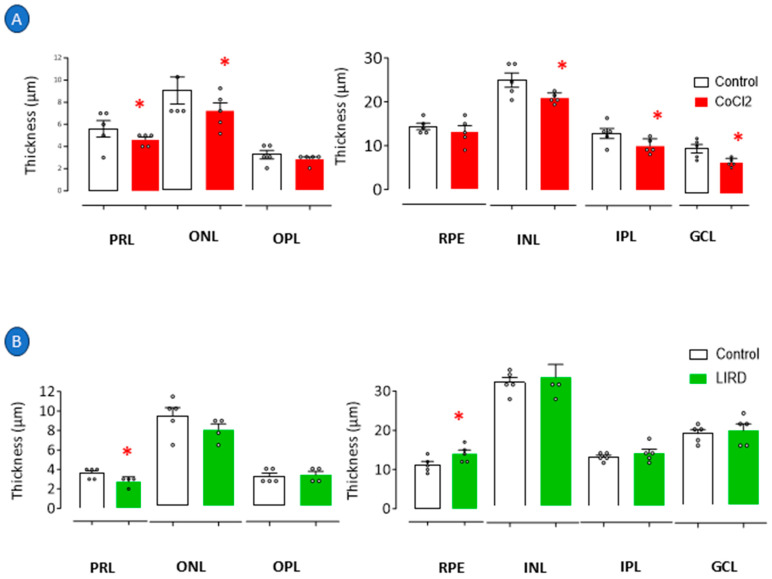
Histologic analysis of retinopathy in zebrafish larvae. Retinopathy was induced in 3 dpf larvae through exposure to E2 0.5x medium at 28 °C with 0.5 mM cobalt chloride (CoCl_2_) for 72 h. Independent group of 3 dpf larvae was exposed to intense light at 8000 lux for 24 h (LIRD). Retinal layer thickness of CoCl_2_-exposed zebrafish larvae (**A**) and zebrafish larvae subjected to LIRD (**B**). * *p* < 0.05 compared to control group. Bars represent mean ± SEM. Dots represent individual data points. PRL: photoreceptor layer; ONL: outer nuclear layer; OPL: outer plexiform layer; RPE: retinal pigment epithelium; INL: inner nuclear layer; IPL: inner plexiform layer; GCL: ganglion cell layer.

To recapitulate the retinal changes induced by hypoxia, we used 3 dpf larvae that were exposed to CoCl_2_ dissolved in a medium for 3 consecutive days (Figure 1A) or 2 dpf larvae that were stimulated by intense light for 24 h after being in the dark for 48 h (Figure 1B). A visual–motor response (VMR) test consisting of exposure to alternating light stimuli was used to evaluate the integrity of the neurological and visual systems of the zebrafish subjected to both protocols. The swimming pattern of the CoCl_2_-exposed larvae demonstrated inertia when compared to embryos from a negative-control group (Figure 1C). Although their locomotion pattern was not altered, exposure to intense light caused a shorter distance to be covered (Figure 1D). Figure 1E shows the quantification of the average total distances covered during the entire VMR period, where exposure to CoCl_2_ resulted in a significant 51% reduction in the total distance traveled relative to the negative controls (*p* = 0.0023), indicating impaired visual–motor function compared to that of the negative-control group. In contrast, light-induced retinal damage (LIRD) led to a 23% decrease in the total distance covered relative to the negative controls (*p* = 0.0171), suggesting a moderate impairment of visual–motor function.

Histological analysis revealed that CoCl_2_-induced damage or LIRD provoked changes in the morphology of the larval retina (Figure 1F), which was characterized by the thinning of the PRL by 17%, ONL by 20%, INL by 16%, IPL by 22%, and GCL by 31% (Figure 2A) compared to those of the negative controls. In the LIRD group (Figure 2B), a 24% decrease in the PRL thickness and a 25% increase in the RPE thickness occurred.

### 2.2. Prophylactic Effects of TnP in Retinopathy Models

**Figure 3:**
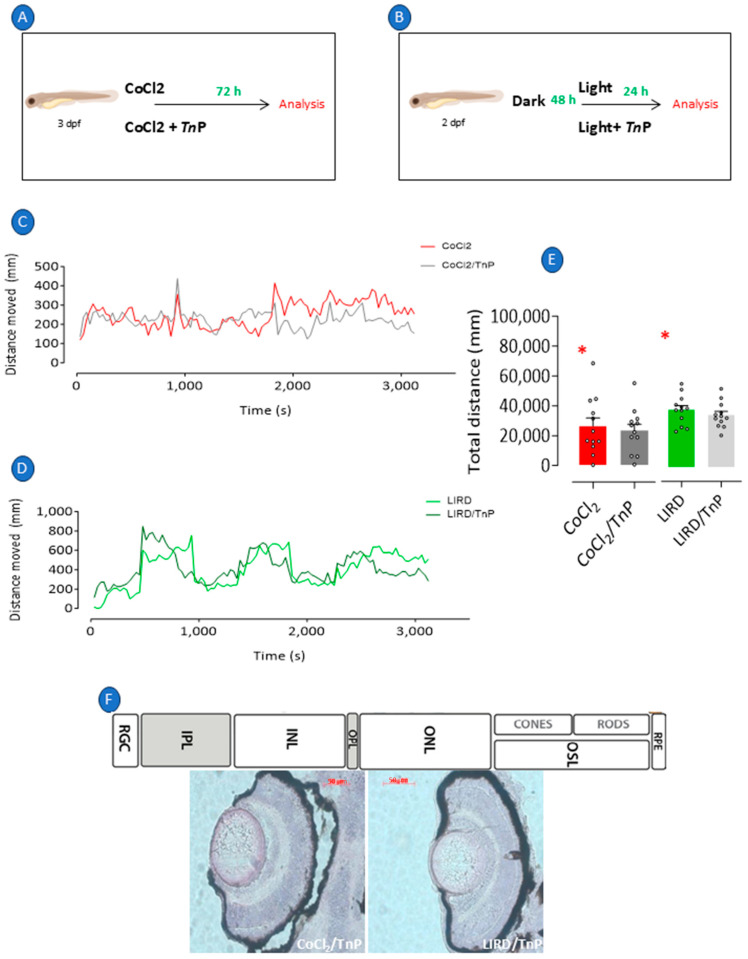
Prophylactic treatment with TnP. Retinopathy was induced in 3 dpf larvae through exposure to E2 0.5x medium at 28 °C with 0.5 mM cobalt chloride (CoCl_2_) for 72 h, and one group was chosen to be prophylactically treated with 100 µM TnP for same period (**A**). Independent group of 3 dpf larvae was exposed to intense light at 8000 lux for 24 h (LIRD), and one group was chosen to be prophylactically treated with 100 µM TnP for same period (**B**). Immediately after stimulation, all groups of larvae were evaluated for their visual–motor response (**C**,**D**) and distance traveled (**E**) and were subsequently killed and processed for histological analysis and H&E staining (**F**). * *p* < 0.05 compared to control group. Bars represent mean ± SEM. Dots represent individual data points. RGL: retinal ganglion layer; IPL: inner plexiform layer; INL: inner nuclear layer; OPL: outer plexiform layer; ONL: outer plexiform layer; OSL: outer segment layer; RPE: retinal pigment epithelium.

When larvae with CoCl_2_-induced damage (Figure 3A) or LIRD (Figure 3B) were treated with TnP during the period of disease development, the treatment did not reverse the reduced total distance traveled presented by the larvae of both models (Figure 3C–E). However, a histological analysis revealed that TnP ameliorated the altered morphology induced by CoCl_2_ or LIRD (Figure 3F).

**Figure 4:**
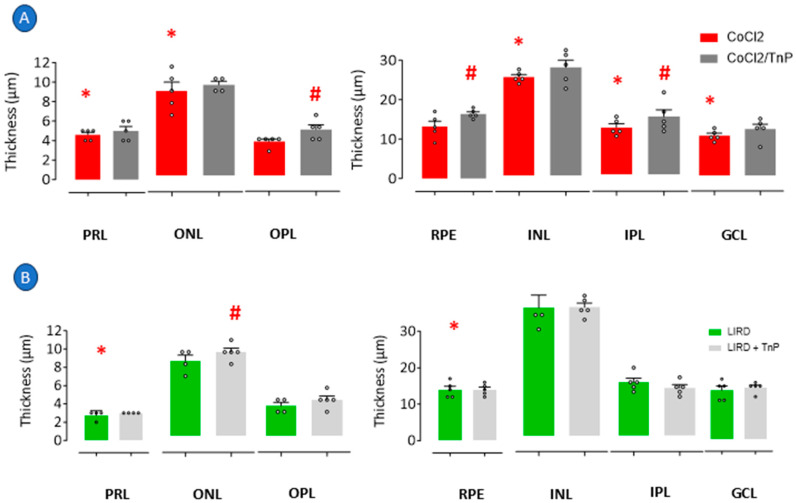
Histologic analysis of retinopathy and prophylactic treatment with TnP. Retinopathy was induced in 3 dpf larvae through exposure to E2 0.5x medium at 28 °C with 0.5 mM cobalt chloride (CoCl_2_) for 72 h, and one group was prophylactically treated with 100 µM TnP for same period. Independent group of 3 dpf larvae was exposed to intense light at 8000 lux for 24 h (LIRD), and one group was prophylactically treated with 100 µM TnP for same period. Retinal layer thickness of CoCl_2_-exposed zebrafish larvae (**A**) and zebrafish larvae subjected to LIRD (**B**). * *p* < 0.05 compared to control group. # *p* < 0.05 compared to disease group. Bars represent mean ± SEM. Dots represent individual data points. PRL: photoreceptor layer; ONL: outer nuclear layer; OPL: outer plexiform layer; RPE: retinal pigment epithelium; INL: inner nuclear layer; IPL: inner plexiform layer; GCL: ganglion cell layer.

In detail, the measurement of the retinal layers of larvae under TnP treatment showed that although the treatment did not reverse the CoCl_2_-induced decrease in the thickness of the PRL, ONL, INL, and GCL, it reversed the thinning of the IPL, inducing an increase in its thickness of 22% in relation to the CoCl_2_-induced group. TnP also induced an increase in the OPL thickness of 35% and the RPE thickness of 24% (Figure 4A). In the LIRD group, TnP did not reverse the thinning of the PRL or increase the thickness of the PRE layer, but it induced the thickening of the ONL by 12% (Figure 4B).

TnP failed to reverse the thinning of all the layers that had been affected by the injury induced through both protocols, as its administration concurrent with the onset of the damage did not appear to impede the process, except for in relation to the IPL, which seemed to be restored. However, our data showed that the outer and inner plexiform layers (OPL and IPL, respectively), where synapses occur and which contain microglia, as well as retinal pigment epithelium (RPE) cells and the photoreceptor rod and cone cell body layer (ONL), could be targets of TnP, and its thickening effects generated improvements in the retina morphology.

### 2.3. Intrinsic Effects of TnP

**Figure 5:**
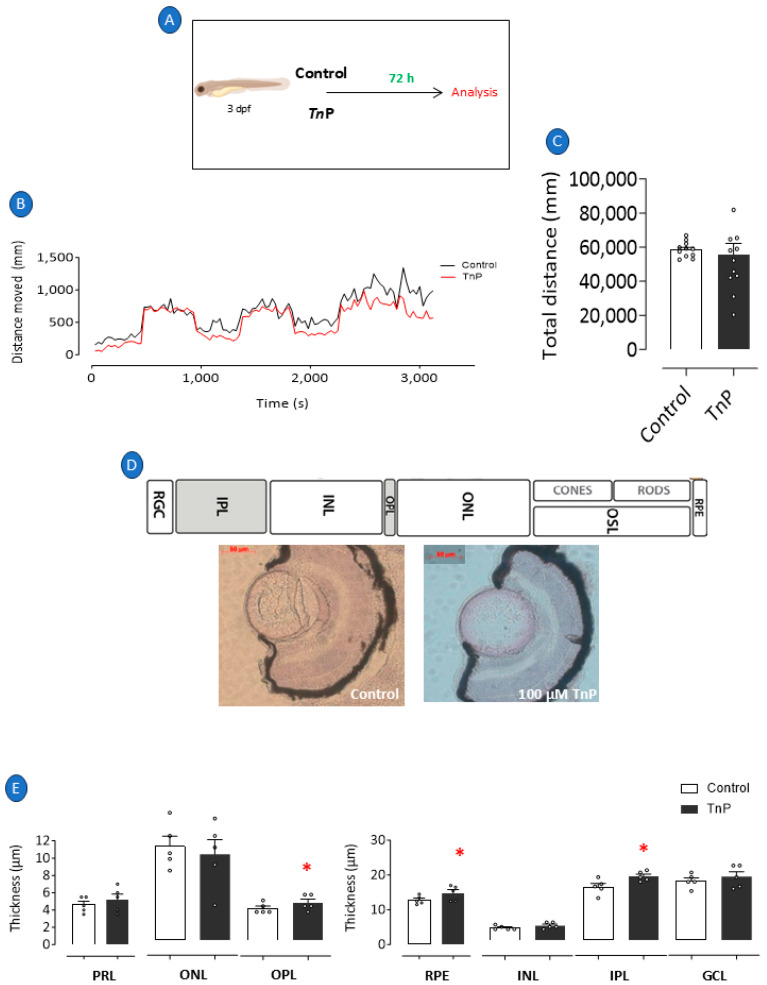
TnP’s per se effects. We prophylactically treated 3 dpf larvae with 100 µM TnP for 72 h (**A**). Larvae maintained at 28 °C in E2 0.5x medium only and under natural light were considered negative controls. Immediately after experimental period, all groups of larvae were evaluated for their visual–motor response (**B**) and distance traveled (**C**) and were subsequently killed and processed for histological analysis and H&E staining (**D**). Retinal layer thickness of zebrafish larvae (**E**). * *p* < 0.05 compared to control group. Bars represent mean ± SEM. Dots represent individual data points. RGL: retinal ganglion layer; IPL: inner plexiform layer; INL: inner nuclear layer; OPL: outer plexiform layer; ONL: outer plexiform layer; OSL: outer segment layer; RPE: retinal pigment epithelium; PRL: photoreceptor layer; GCL: ganglion cell layer.

A cohort was formed by subjecting 3 dpf larvae to TnP throughout the experimental period to investigate the intrinsic effects of TnP on retinal cells, as depicted in Figure 5A. Notably, the group treated exclusively with TnP exhibited no significant difference in the total distance traveled compared to the control group, as shown in Figure 5B,C. However, after 72 h of TnP exposure, there was a substantial increase in the thickness of the OPL by 18%, the RPE by 16%, and the IPL by 20%, as illustrated in Figure 5E and the photomicrograph in Figure 5D.

### 2.4. Antiangiogenic Effects of TnP

**Figure 6:**
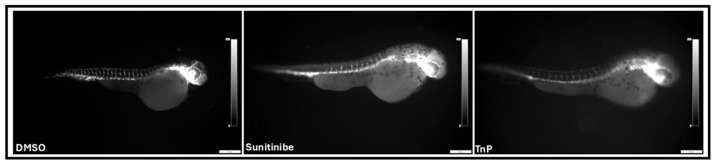
Antiangiogenic activity of TnP. Fluorescence images of intersegmental vessels in 0.15% DMSO vehicle control group, 10 µM sunitinib-exposed group (SU 10), and 100 µM TnP-treated group (TnP). Missing and incomplete vessels were evident in 10 µM sunitinib-exposed group (SU 10) and 100 µM TnP-treated group (TnP).

**Figure 7:**
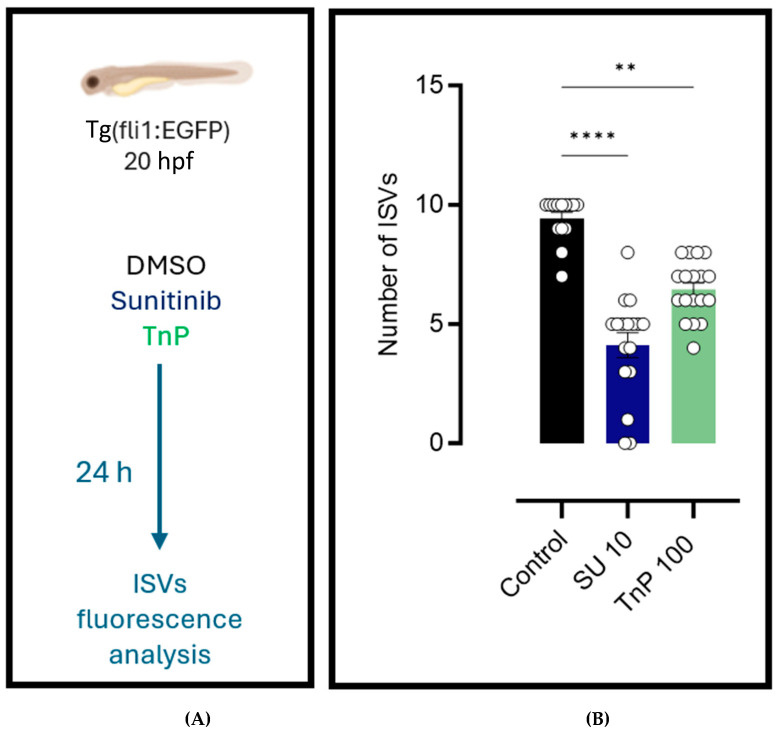
Antiangiogenic activity of TnP. (**A**) Tg(fli1:EGFP) zebrafish larvae (20 hpf) were dechorionated and sorted into 3 groups subjected to distinct treatments for 24 h: the 0.15% DMSO vehicle control group, 10 µM sunitinib-exposed group (SU 10), and 100 µM TnP-treated group (TnP). (**B**) The number of intersegmental vessels (ISVs). ** = *p* = 0.0011, **** = *p* < 0.0001, compared to the control group.

The fluorescence microscopy of the intersegmental vessels in zebrafish larvae from the Tg(fli1:EGFP)y^1^ line (Figure 6) revealed that treatment with TnP (100 µM) and sunitinib (10 µM) exposure significantly affected vascular development compared to the vehicle control group (0.15% DMSO). At 44 h post-fertilization (hpf), the larvae treated with TnP exhibited a marked reduction (60%; *p* = 0.0011) in the number of intersegmental vessels in the trunk region, between the fourth and fourteenth somites, when compared to the control group (Figure 7).

Quantitative analysis showed that the larvae exposed to TnP displayed a vessel count similar to that of the larvae treated with sunitinib. Specifically, both treatments resulted in a decrease in the number of intersegmental vessels compared to that in the control group. The reduction in the vessel number in both the treatment groups was statistically significant, indicating that TnP promotes antiangiogenesis, similarly to sunitinib.

## 3. Discussion

### 3.1. Modulation of Retinal Degeneration and Regeneration: Effects of TnP in Hypoxia and LIRD Models

Retinal hypoxia is a key factor in diabetic retinopathy, contributing to vascular occlusion [20]. In zebrafish, hypoxia can be modeled using cobalt chloride (CoCl_2_), which stabilizes HIF-1α and induces endoplasmic reticulum (ER) stress [21,22]. Alternatively, light-induced retinal degeneration (LIRD) selectively damages photoreceptors, followed by robust regeneration by stem cells [23].

Our data show that LIRD causes milder initial damage than CoCl_2_ exposure. The lack of a locomotor response in the CoCl_2_-treated larvae likely reflects widespread retinal cytotoxicity, impairing light perception. In contrast, LIRD larvae retained some visual function, enabling light-dependent movement under scotopic conditions. This suggests the partial preservation of photoreceptor activity despite the injury.

Both models effectively induced retinal damage, though through distinct mechanisms. CoCl_2_ caused broad retinal injury, affecting photoreceptors, inner nuclear layer (INL) cells (amacrine, bipolar, horizontal, and Müller glia), and ganglion cells. LIRD primarily targeted photoreceptors and the retinal pigment epithelium (RPE). Histological analysis revealed the thinning of the outer nuclear layer (ONL) and photoreceptor layer (PRL), consistent with photoreceptor loss.

The retina’s high metabolic demand makes it vulnerable to oxidative stress, which triggers inflammation [24]. CoCl_2_ mimics hypoxia, promoting a pro-inflammatory environment that leads to apoptosis and cellular senescence [22,25]. Similarly, LIRD upregulates inflammatory mediators like IL-1β, TNF-α, and COX-2 [26,27,28,29], alongside promoting photoreceptor apoptosis and RPE vacuolization [24,29]. Anti-TNF-α therapies are already used clinically for ocular inflammation, underscoring the role of inflammation in retinal pathology.

TnP has previously demonstrated anti-inflammatory effects in experimental autoimmune encephalomyelitis (EAE), reducing microglial activation, cytokine production, and leukocyte infiltration [8]. Given its neuroprotective potential, we hypothesized that TnP could mitigate retinal degeneration. Indeed, TnP treatment increased the thickness of the synaptic layers (OPL, IPL) and the RPE, suggesting partial recovery from inflammation-induced damage.

In cuprizone-treated mice, TnP restored their axonal health [8]. Here, TnP improved the retinal structure in CoCl_2_-injured larvae, particularly in the OPL, RPE, and IPL, without affecting locomotor activity. These findings align with its proposed role in the CNS and retinal repair.

TnP also exhibited mild antiangiogenic effects, though they were less potent than those of sunitinib. By inhibiting abnormal vascular growth, TnP may help restore retinal homeostasis. Utilizing a VEGF blockade is a common strategy in treating diabetic retinopathy and AMD, as it reduces pathological neovascularization [30]. Additionally, TnP may modulate integrins (e.g., αVβ3, αVβ5), which regulate angiogenesis and inflammation by influencing cell–matrix interactions.

Inflammation also activates Müller glia, which in zebrafish can dedifferentiate into progenitor cells to aid regeneration [31]. In mammals, however, Müller cells typically undergo reactive gliosis, limiting recovery. Pathways like the Wnt/β-catenin pathway and Slit proteins may promote a regenerative phenotype, offering therapeutic targets. Combining VEGF inhibition with glial and immune modulation could enhance retinal repair while minimizing the side effects.

### 3.2. Study Limitations

While this study highlights TnP’s protective effects in retinal degeneration models, several limitations should be noted:

1. Molecular mechanisms: Gene expression or receptor interaction studies were not performed. Future work should incorporate a PCR, RNA-seq, or proteomics to elucidate TnP’s pathways.
2. Functional assessment: The visual–motor response (VMR) was the sole behavioral metric. Complementary tests (e.g., optokinetic response tests, electroretinography) would strengthen the functional correlations.
3. Comparator drugs: The absence of a standard anti-inflammatory control limited direct efficacy comparisons. Benchmarking against established therapies (e.g., anti-VEGF agents) is needed.
4. Mechanistic insights: TnP’s proposed actions (e.g., integrin/VEGF modulation) remain hypothetical. Molecular docking, binding assays, and signaling studies are essential for their validation.

Despite these gaps, our findings support TnP being a promising candidate for retinal therapy, warranting further mechanistic and translational investigation.

## 4. Materials and Methods

### 4.1. TnP Peptide

A TnP trifluoroacetate compound (C63H114N22O13S4, 97.3% purity) was synthetized in the solid phase and purchased from GenScript (#P13821401, Piscataway, NJ, USA). TnP solutions were prepared by diluting lyofilizated powder in an E2 0.5x medium (7.5 mM KH_2_PO_4_, 2.5 mM Na_2_HPO_4_, 15 mM NaCl, 0.5 mM KCl, 1 mM MgSO_4_·7H_2_O, 1 mM CaCl_2_·2H_2_O, 0.7 mM NaHCO_3_).

### 4.2. Zebrafish Husbandry

Adult zebrafish (<18 months old) belonging to the AB strain (International Zebrafish Resource Center, Eugene, OR, USA) were kept separated by their sex and maintained under standard conditions at 28 °C and pH 7 and under a light–dark cycle of 14/10 h in individual aquariums in an ALESCO (ALESCO, Campinas, Brazil) rack using system water (60 μg.mL^−1^ Instant Ocean Sea salts). The experiments were carried out following the regulations of the National Council for Animal Experiment Control (CONCEA) and approved by the Butantan Institute’s Ethics Committee on the Use of Animals (CEUAIB #7930250423). The fertilized embryos checked using a Leica EZ4W stereomicroscope (Leica Microsystems, Cambridge, UK) were transferred to 100 × 25 mm plastic dishes (89107-632, VWR) containing an E2 0.5x medium (7.5 mM KH_2_PO_4_, 2.5 mM Na_2_HPO_4_, 15 mM NaCl, 0.5 mM KCl, 1 mM MgSO_4_·7H_2_O, 1 mM CaCl_2_·2H_2_O, 0.7 mM NaHCO_3_) and classified according to Kimmel et al. (1995) [32].

### 4.3. Zebrafish Anesthesia and Euthanasia

Anesthesia was performed by immersing the larvae in 2 mL of an E2 0.5x medium containing 0.4% tricaine (ethyl-3-aminobenzoate, #MS-222, Sigma Chemical Co., St. Louis, MO, USA) for 2 min at room temperature before analysis. At the end of the experiments, euthanasia was performed through immersion in 4% tricaine diluted in an E2 0.5x medium. After exposure, the larvae were checked in an M205C stereomicroscope (Leica Microsystems) to ensure that their hearts were not beating.

### 4.4. Cobalt Chloride-Induced Retinopathy and TnP Prophylactic Treatment

Zebrafish larvae at 3 dpf (n = 30/group) were exposed in an E2 0.5x medium to cobalt chloride II hexahydrate (CoCl_2_, #255599, Sigma Aldrich, São Paulo, Brazil) at 0.5 mM for 72 h (CoCl_2_ group). Treatment with TnP at 100 μM was carried out by adding it to the E2 0.5× medium concomitantly with cobalt (CoCl_2_/TnP group). An independent group of larvae was exposed to TnP alone for 3 days (TnP group). Larvae left in only an E2 0.5x medium and untreated were considered negative controls (control group). Every 24 h the E2 0.5x medium was changed and cobalt and TnP were re-added. All groups were incubated at 28 °C. At the end of the experiments, a visual–motor response test was performed, followed by euthanasia, and samples were collected for histological analysis.

### 4.5. Intense Light-Induced Retinal Damage and TnP Prophylactic Treatment

To induce retinopathy with intense light, independent groups of 2 dpf larvae (n = 30/group) were left in a 0.5x E2 medium for 48 h in the dark and then exposed to light (8000 lux) for 24 h (LIRD group). A group of larvae was maintained in an E2 0.5x medium for two periods of 48 h of darkness and 24 h more of intense light (8000 lux) in the presence of TnP at 100 μM (LIRD/TnP group). Larvae exposed to a normal photoperiod were considered negative controls (control group). Every 24 h the E2 0.5x medium was changed. All groups were incubated at 28 °C. At the end of the experiments, a visual–motor response test was performed, followed by euthanasia, and samples were collected for histological analysis.

### 4.6. Visual–Motor Response

This test consisted of exposure to 8 periods alternating between light and dark of 450 s. Locomotor activity was investigated by evaluating the swimming behavior during the dark–light transition at the end of each experiment. The total displacement was evaluated in a Zebrabox system (ViewPoint Life sciences, Lyon, France), and the locomotor activity was quantified and analyzed using ZebraLab™ version 3.52 (Pisa, Italy) by ViewPoint. The mean velocity was adjusted to values between 1.8 and 4.0 mm/s, while any movement slower than 1.8 mm/s was considered inactive and above 4.0 mm/s was considered agitated behavior. The results for the total distance traveled were obtained by adding the distances traveled at medium and agitated speeds.

### 4.7. Hematoxylin/Eosin (H&E) Histological Analysis

Whole zebrafish larvae were washed with PBS, fixed in 4% formaldehyde in PBS overnight, and then dehydrated with different concentrations of 30–70% methanol (MeOH) diluted in PBST (50 mL 10× PBS, 1 mL 10% Tween 20, increased to a volume of 500 mL with dH_2_O) for 10 min each and during agitation. The next day, the larvae were rehydrated by washing them sequentially with 70–30% MeOH diluted in PBST for 10 min each during agitation, followed by two sequential washes for 5 min with PBST during agitation. After the removal of the PBST, the samples were transferred to a 30% sucrose solution and kept overnight. For cryosection, 3 fixed larvae were assembled in an OCT (optimal cutting temperature compound) cryomold (total of 4 cryomolds per group), frozen and sectioned at a thickness of 18 μm, in a coronal position, in a cryostat (Cryostat Leica CM1860), and processed for H&E staining. All the slides were examined through optical microscopy at a 40× magnification (Axio Imager A1, Carl Zeiss, Oberkochen, Germany). Slides containing 8 sections of the assembled groups of 3 larvae were stained and examined in ImageJ Software (U.S. National Institutes of Health, Bethesda, MD, USA, version 1.53k) by measuring the thickness (μm) of the ganglion cell layer (GCL), inner plexiform layer (IPL), inner nuclear layer (INL), outer plexiform layer (OPL), outer nuclear layer (ONL), photoreceptor layer (PRL), and retinal pigment epithelium (RPE).

### 4.8. Vasculature Assay

Zebrafish larvae from the transgenic Tg(fli1:EGFP)y^1^ line, in which endothelial cells express enhanced green fluorescent protein (EGFP), were used to visualize and quantify the intersegmental vessels. The positive control was the potent angiogenesis inhibitor sunitinib (purchased from Merck; code: PZ0012) [20]. The larvae were maintained under controlled conditions with a constant temperature of 28.5 °C and a 14/10 light/dark cycle. At 20 h post-fertilization (hpf), the larvae were dechorionated and assigned to three experimental groups: the vehicle control (0.15% DMSO), SU10 (exposed to 10 µM sunitinib), and TnP (treated with 100 µM TnP) groups. The larvae were kept under these conditions for 24 h, after which they were fixed in 4% formaldehyde for the visualization of the intersegmental vessels.

Fluorescence imaging was performed using an Olympus SZX16 stereomicroscope (Tokyo, Japan) equipped with an SZX2-ILLT LED transmitted light base and fluorescence filter sets. This system allowed for the high-resolution imaging of whole-mount zebrafish larvae and tissues, offering a wide field of view with a maximum optical resolution of approximately 5–10 µm, suitable for capturing gross morphological changes, lesion areas, and fluorescence signals at the tissue level. The fluorescence microscopy system was equipped with an appropriate filter set for EGFP excitation and emission. For each larva, images were captured in the trunk region, between the fourth and fourteenth somites, in order to standardize the analysis. The intersegmental vessels were manually quantified [21]. This protocol enabled a detailed examination of the morphology and integrity of the intersegmental vessels in Tg(fli1:EGFP)y^1^ zebrafish larvae, allowing for a comparative analysis across the different experimental conditions.

### 4.9. Statistical Analysis

All values were expressed as the mean ± the SEM. The experiments were performed independently two times. Parametric data were evaluated using an analysis of variance, followed by the Bonferroni correction for multiple comparisons. Non-parametric data were assessed using the Mann–Whitney test. Differences were considered statistically significant at *p* < 0.05, with the significance determined using GraphPad Prism (Graph Pad Software, v6.02, 2013, La Jolla, CA, USA).

## 5. Conclusions

These findings highlight the importance of developing complementary strategies to support retinal recovery and restore visual function after injuries. The differences in locomotor responses between the experimental groups suggest distinct damage mechanisms, which may require tailored therapeutic approaches. A deeper understanding of these processes could enable more targeted interventions for specific retinal layers. Furthermore, our results validate the use of zebrafish larvae as a valuable model for studying visual–motor deficits in retinopathy and demonstrate how CoCl_2_- and LIRD-induced injuries produce distinct behavioral outcomes.

Histological analysis revealed the cellular basis of these functional differences. CoCl_2_ exposure caused widespread degeneration, affecting both photoreceptors and inner retinal neurons (e.g., bipolar and ganglion cells), while LIRD primarily damaged the photoreceptor layer and retinal pigment epithelium (RPE). These observations clarify how retinal structure and function are disrupted in different injury models.

Using zebrafish larvae, we evaluated TnP’s therapeutic potential in two retinal degeneration models. TnP treatment attenuated the damage in both, particularly by preserving or restoring structurally critical layers. These protective effects may have stemmed from reduced cellular stress and enhanced regenerative processes, such as proliferation or axonal recovery. Although preliminary, these results position TnP as a promising candidate for safeguarding the retinal structure and function.

Notably, TnP may also modulate neurons in the inner nuclear layer, potentially improving the synaptic connectivity between bipolar and amacrine cells. Such plasticity could aid functional recovery, given these cells’ role in retinal signal transmission. However, further research is needed to elucidate TnP’s cellular-level mechanisms, especially in retinal neurons.

Collectively, our findings suggest that TnP influences multiple pathways, including those for inflammation, angiogenesis, and retinal homeostasis signaling. Unraveling these mechanisms could guide future therapies. While TnP shows promise as a neuroprotective and immunomodulatory agent, combining it with other treatments may optimize its therapeutic potential.

## Data Availability

The original contributions presented in the study are included in the article, further inquiries can be directed to the corresponding author.
